# Pathogenicity and virulence of *Shigella sonnei*: A highly drug-resistant pathogen of increasing prevalence

**DOI:** 10.1080/21505594.2023.2280838

**Published:** 2023-11-23

**Authors:** Xosé M. Matanza, Abigail Clements

**Affiliations:** Centre for Bacterial Resistance Biology, Department of Life Sciences, Imperial College London, London, UK

**Keywords:** *Shigella sonnei*, diarrhoea disease, bacillary dysentery, bacterial pathogenesis

## Abstract

*Shigella* spp. are the causative agent of shigellosis (or bacillary dysentery), a diarrhoeal disease characterized for the bacterial invasion of gut epithelial cells. Among the 4 species included in the genus, *Shigella flexneri* is principally responsible for the disease in the developing world while *Shigella sonnei* is the main causative agent in high-income countries. Remarkably, as more countries improve their socioeconomic conditions, we observe an increase in the relative prevalence of *S. sonnei*. To date, the reasons behind this change in aetiology depending on economic growth are not understood. *S. flexneri* has been widely used as a model to study the pathogenesis of the genus, but as more research data are collected, important discrepancies with *S. sonnei* have come to light. In comparison to *S. flexneri, S. sonnei* can be differentiated in numerous aspects; it presents a characteristic O-antigen identical to that of one serogroup of the environmental bacterium *Plesiomonas shigelloides*, a group 4 capsule, antibacterial mechanisms to outcompete and displace gut commensal bacteria, and a poorer adaptation to an intracellular lifestyle. In addition, the World Health Organization (WHO) have recognized the significant threat posed by antibiotic-resistant strains of *S. sonnei*, demanding new approaches. This review gathers knowledge on what is known about *S. sonnei* within the context of other *Shigella* spp. and aims to open the door for future research on understanding the increasing spread of this pathogen.

## Introduction

Diarrhoea is a major life-threatening concern that strongly affects children under 5 years of age. It accounts for 525,000 annual deaths in this age group, mostly occurring in countries under economic development [1,2]. Shigellosis, also known as bacillary dysentery, is the disease caused by species of the genus *Shigella* when they invade and damage the colonic epithelium. Clinical symptoms of the disease can vary from mild, self-limiting diarrhoea to severe diarrhoea with blood in stools and other complications [3,4]. Noteworthy, according to the Global Enteric Multicentre Study (GEMS), *Shigella* is the most common pathogen associated with diarrhoea in children aged 2–5 years in low-income countries [5]. Bacillary dysentery is the second cause of diarrhoeal mortality, occasioning 212,438 deaths annually of which 30% are children under 5 years of age [6]. Exposure to *Shigella* has also been associated with long-term growth problems and neurological development delays in children in low-income countries [7,8].

Belonging to the family *Enterobacteriaceae*, the genus *Shigella* are Gram-negative, facultatively anaerobic, rod-shaped, non-motile bacteria that classify into four species: *Shigella flexneri*, *Shigella sonnei*, *Shigella dysenteriae* and *Shigella boydii* [9]. The current method of classifying the genus is based on serology, by the analysis of their O-antigen type. This divides *Shigella* into groups or species: group A, or *S. dysenteriae*, with 15 serotypes; group B, or *S. flexneri*, with 19 serotypes and subserotypes; group C, or *S. boydii*, with 20 serotypes; and as an exception, group D or *S. sonnei*, which presents only one serotype [10].

*Shigella* spp. differ extensively in their geographical distribution and their contribution to the burden of disease. Currently, *S. dysenteriae* and *S. boydii* are seldom isolated, while *S. flexneri* and *S. sonnei* contribute to 90% of all cases [5]. In low-income countries in Africa, Asia and South America, the main burden of shigellosis is infection by *S. flexneri*, although approximately 25% of cases are still caused by *S. sonnei* [11,12]. In contrast, for the bulk of cases in wealthy countries, predominantly in Europe and North America, the predominant aetiological agent is *S. sonnei* [13]. Remarkably, global socioeconomic improvements are leading to a shift in shigellosis causative agents, with *S. sonnei* replacing *S. flexneri* in many diagnoses in transitional countries [14].

The pathobiology of *Shigella* spp. has traditionally been studied using *S. flexneri* as the primary model, but assumptions based on this organism are insufficient to explain the findings of *S. sonnei* research. As such, further investigations specifically targeting the latter are crucial. This review aims to provide an overview of the biology and virulence of *S. sonnei* against the backdrop of other *Shigella* spp., summarizing the current knowledge and pinpointing areas for future study.

## Epidemiology

Among the four species of *Shigella*, S. *dysenteriae* is considered the most severe and has provoked four pandemics in the second half of the 20th century in America, Africa, and Asia [15,16]. The high virulence profile is associated with the production of the Shiga-toxin Stx [17]. *Shigella boydii* is acknowledged to be restricted to Bangladesh, India, Nigeria, Yemen, and other South-Eastern Asian countries; this species hardly affects outside these territories except for travel-associated episodes [18]. *S. flexneri* predominantly causes episodes of shigellosis in low-income countries with inadequate hygienic and sanitary conditions, where problems such as poor water sanitation and malnutrition are common. *S. flexneri* is accountable for 62% and *S. sonnei* for 25% of shigellosis cases in Asia and sub-Saharan Africa [19]. However, as hygiene conditions and the economy of the country grows, *S. sonnei* causes the highest number of cases. 80% of shigellosis episodes across Europe and North America result from *S. sonnei* infections [13,19–21]. A current issue that demands closer examination is the fact that as more countries industrialize, *S. sonnei* is surpassing *S. flexneri* as the aetiological agent of disease [14]. An eight-year analysis conducted between 2004 and 2011 in Beijing (China) showed how *S. sonnei* has replaced *S. flexneri* as most prevalent since 2009 [22]. In Vietnam, throughout a period of 14 years (1995–2008) *S. sonnei* cases increased from 29% to the 78% of *Shigella* isolations [23]. In Bangladesh, *S. sonnei* increased from around 10% of all *Shigella* cases in 2001 to nearly 50% in rural areas and over 50% in urban settings by 2020 [24]. More countries experiencing this phenomenon include Brazil [25], India [26], and Iran [27], among others. In fact, country gross domestic product (GDP) positively correlates with an increased isolation of *S. sonnei* as the main cause of shigellosis [28].

Improvements in sanitation standards are accompanied by a reduced risk of diarrhoeal diseases, however this does not entirely apply for shigellosis as it appears to not fully extend to *S. sonnei*. The reason for this shift in the aetiological agent linked to economic and healthcare prosperity is not completely understood, although various hypotheses have been under consideration [14]. One hypothesis was the utilization of amoebae as protective hosts that could increase *S. sonnei* survival in the environment. Amoebae can be encountered in numerous aquatic (sea, lakes) and terrestrial (soil, dust) niches, but they have also been found to resist water chlorination in drinking water and pools [29]. While amoebae are known to predate on bacteria, some bacterial species have evolved defensive tactics to either replicate within the amoebic cell or live alongside them as a saprophyte [30]. Examples of these associations include *Legionella pneumophila*, *Francisella tularensis*, *Vibrio cholerae*, *Mycobacterium leprae* or *Chlamydia pneumoniae* among others [31–33]. However, the theory suggesting that *S. sonnei* might use amoebae to enhance its survival even in places with good living conditions has proved false; *S. sonnei* and *S. flexneri* survive equally in long-term cultures with amoebae, but neither species can survive inside the protozoan cells [34].

One of the most compelling explanations to explain the differences between *S. flexneri* and *S. sonnei* geographical distributions has to do with the O-antigen component of lipopolysaccharide (LPS) of the bacterium. The genetic machinery of O-antigen biosynthesis and export in *Shigell*a is mostly located on the chromosome with minor exceptions [35,36]. For instance, some *S. flexneri* encode a second *wzz* co-polymerase on the plasmid pHS-2 [36]. *S. sonnei* diverges from this pattern; the chromosomal locus is disrupted, and the O-antigen cluster is instead harboured on a large virulence plasmid (LVP). The O-antigen locus was acquired via horizontal transfer from the distant gram-negative bacterium *Plesiomonas shigelloides* [35,37,38]. *P. shigelloides* is commonly found in contaminated water in low-income countries which may provide natural protection against *S. sonnei* [37,39]. Conversely, in resource-rich or transitional countries that improve water sanitation, where people have little contact with *P. shigelloides*, cross-immunity cannot occur and *S. sonnei* may emerge as the primary cause of shigellosis. Further data collection is required to verify whether exposure to a single serotype of *P. shigelloides* (O17) occurs at sufficient levels to protect the population against *S. sonnei* infections.

## Key aspects in the evolution of S*higella* spp

*Shigella* spp. are specialized lineages arisen from different *E. coli* ancestors that evolved convergently. Advances in genome sequencing have enabled the classification of *Shigella* spp. into three genomic clades, where serotypes distribute without clade-species correlation. This implies that the conventional serological classification of *Shigella* does not agree with their evolutionary history. In addition to these three clusters, there are several outliers that contain some members of *S. dysenteriae*, *S. boydii* and all those belonging to *S. sonnei*. Confirming the evolutionary relationship between *Shigella* spp. and *E. coli*, all clusters and outliers intersperse with *E. coli* members [10,40]. Phylogenomic analyzes underscore clade differentiation within *Shigella* [41].

*Shigella* and *E. coli* share a core genome of around 3 Mb, however; insertions, deletions, and genetic rearrangements operating in different ancestors have led to a reduction of genome size, accumulation of pseudogenes and loss of biological functions, especially in *Shigella* [42,43]. Insertion sequences are key to comprehend the evolution of *Shigella* and many other well-adapted human pathogens [44–47]. Compared to other bacterial species, *Shigella* genomes contain the highest number of insertion sequences relative to their size [42,48,49]. Owing to the effect of insertion sequences, some core metabolic pathways present in *E. coli* members are absent in *Shigella* species. For example, *Shigella* spp. are unable to ferment lactose due to various alterations in the lac operon [50]. Insertion sequences also inhibit the utilization of tryptophan as a carbon and nitrogen donor [51] and stop conversion of the amino acid lysine into cadaverine due to the mutation of the lysine decarboxylase gene *cadA* [52]. *cadA* is considered an antivirulence gene in *Shigella* that has undergone a high evolutionary pressure to be eliminated from the genome of these species. When *cadA* is expressed at normal levels (or cadaverine is present), cell invasion and evasion of host immune defences are impaired [53]. In addition, one of the main phenotypical characteristics of *Shigella*, i.e. the absence of flagellar motility, comes from the accumulation of mutations and insertion sequences in different genes and has been related to reduced recognition of the pathogen by the host immune system [42,54,55]. Insertion sequences operate in the progressive reduction of the genomes of *S. flexneri* and *S. sonnei*, resulting in smaller genomes that resemble the hyper-reduced genome of *S. dysenteriae*. Interestingly, mutations in genes that have already disappeared in *S. dysenteriae* are emerging in *S. flexneri* and *S. sonnei* clones [49]. When certain biochemical pathways are lost in a parallel pattern, it suggests that their absence confers an adaptive advantage to exploit resources in a new environment [52]. In the case of *Shigella* spp., these convergent alterations that lead to a smaller genome could be improving adaptation to the human host and contributing to pathogenicity.

Horizontal gene transfer is another key phenomenon that explains *Shigella* spp. evolution. The acquisition of the large virulence plasmid (LVP) pINV on multiple occasions by various *E. coli* founder ancestors was a crucial factor that gave rise to major *Shigella* lineages. The plasmid has an approximate length of 220 Kb and harbours a particularly important locus for *Shigella* pathogenicity, the 30 Kb *ipa*–*mxi*–*spa* region that encodes a type 3 secretion system (T3SS) along with regulatory proteins and effectors [56]. The LVP gives *Shigella* the capacity to invade host cells and alter the host immune system [15]. This plasmid can be found in two mutually incompatible isoforms, A and B, defined by the sequence of genes *mxiA*, *mxiC*, and *IpgD* [57]. Isoform A is present in clade 1, Isoform B in clade 3 and both in clade 2 [57,58]. Outliers to the clades may harbour either of the two versions [59]. *S. sonnei* harbours isoform B, and the LVP additionally encodes for the *P. shigelloides* serogroup 17 O-antigen and has shaped the epidemiology and emergence of this species [35]. Notably, pINV loss is very frequent and higher in *S. sonnei* compared to *S. flexneri*. This disparity is attributed to the removal of toxin-antitoxin systems facilitated by the presence of insertion sequences in *S. sonnei* [60,61].

*S. sonnei* originated more recently than the rest of *Shigella* from a common European ancestor, estimated to be less than 500 years old. This ancestral strain underwent subsequent expansion and diversification, leading to the formation of four distinct lineages (Lineage I to Lineage IV). Notably, Europe exhibits a higher level of genetic diversity with representatives of the four lineages [13,35]. Lineage III has exhibited a higher level of success in terms of its global spread, accounting for most strains present in South America, Africa, and Asia [13,14,62,63]. One of key characteristics of lineage III is its higher accumulation of resistances to antibiotics, a fact that is key for its global expansion [55,64].

## Shigellosis

### Transmission

Humans constitute the only natural reservoir for *Shigella* species [3]. One of the key factors contributing to their high spread is their very low infective dose, with as few as 10–100 being enough to cause disease [65]. As a result of such small inoculum, the principal way *Shigella* transmits is through the faecal-oral route from person-to-person. Infections are more frequent in regions or environments without safe conditions of hygiene and water potability [3]. *Shigella* can also be spread by swallowing water that has been in contact with infected faecal matter [66–68] and is considered a foodborne pathogen, especially associated with unsuitable food-handling practices [69,70]. The clinical management of food and waterborne outbreaks may be complicated because of secondary person-to-person dissemination [70,71]. Propagation may also take place through flies as a mechanical vector [72]. Recent *Shigella* outbreaks have been transmitted during sexual activity through contact with an infected person’s excrement [73,74].

### Symptomatology

All species of *Shigella* cause a similar range of symptoms, although *S. dysenteriae* type 1 infections are the most severe [9,75]. *S. sonnei* infections are often described as less severe than those caused by *S. flexneri* [76] but clinical comparisons indicate similar symptom severity [77,78]. The time interval between infection and becoming symptomatic is usually 1 to 4 days, although it can extend to 8 days in the case of *S. dysenteriae* [79]. In some individuals, no symptoms are present, which is crucial for the transmission of the pathogen [80]. In general, in countries with higher resources, while individuals with shigellosis may experience severe symptoms, the illness typically resolves within a short time period. In contrast, in lower resource countries shigellosis is linked with increased levels of morbidity and mortality [4,9,81].

Most infections produced by *Shigella* spp. cause fever, vomiting, watery diarrhoea, cramps, and abdominal pain [3]. When cases evolve in severity, the presence of blood and/or mucus in faeces (a clinical manifestation of gut epithelial damage) and tenesmus may appear. Patients can also show a wide variety of complications such as intestinal perforation and obstruction, rectal prolapse, recurrent diarrhoea, dehydration, seizures, vaginitis and encephalopathy among others [3,82,83]. Complications have been reported to occur in infections with any *Shigella* species [23,75]. Young age, HIV immunodeficiency and malnutrition are regarded as risk factors, however it is not a prerequisite since complications may be observed in apparently healthy individuals [3,84].

### Groups at higher risk

#### Children under 5 years of age in low- and middle- income countries

*Shigella* infections are one of the major life-threatening hazards to which children from low- and middle- income countries, particularly in sub-Saharan Africa and South Asia, are exposed [3,23,24,82,85]. *Shigella* is the most prevalent cause of diarrhoeal illness among children between the ages of two and five, and the second most common cause in children below two years of age [86]. The true burden of child shigellosis in low-resource settings is often underestimated. The World Health Organization (WHO) recommends antibiotic administration for children only when dysentery (bloody depositions) is observed. However, dysentery by itself is not an accurate predictor of *Shigella* since many fatal infections occur without this symptom [87]. Therefore, antibiotic administration to particularly vulnerable children without dysentery should be considered in order to minimize the risk of complications [85,87,88].

### Children in day-care centres and crowded settings

In high-income nations, children are also particularly targeted by the disease. The close contact between children as well as their poor toileting skills facilitate *Shigella* outbreaks in day care centres, schools, and other crowded settings. Secondary cases affecting other members of the family or the community are common [89–92]. As an example, in northwest Missouri almost half of the cases of shigellosis reported from May to October 2005, occurred in children in day-care centres, their families or people working in these centres [90].

### Men who have sex with men (MSM)

Men who have sex with men (MSM) are at a higher risk of contracting shigellosis due to the increased likelihood of direct or indirect faecal-oral contact during sexual activity [93]. Although *S. flexneri* serotypes 2a and 3a account for numerous cases, the majority of sexually transmitted *Shigella* infections are caused by *S. sonnei* [20,73]. In recent years, multidrug resistant *S. sonnei* has caused multiple outbreaks among MSM in European countries [94–98], Australia [74] and in the United States [99]. According to recent data, the incidence of shigellosis among MSM in England has almost doubled during the last 10 years, with more than 90% of all isolates showing high resistance to multiple antibiotics [100]. The existence of recurrent and persistent infections among MSM highlights the need for new strategies to tackle the infection [101].

### Persons experiencing homelessness (PEH)

People experiencing homelessness (PEH) face conditions that may predispose for contracting shigellosis both sleeping outdoors or when in shelters i.e. high infectious dose, exposure to contaminated food and water, overcrowding, drug use and poor access to hygiene and sanitation [102–104]. An increasing number of highly antibiotic resistant outbreaks of shigellosis, mostly caused by *S. sonnei*, affecting PEH in HIC has been described in recent years [78,102,103,105–107]. Over half of the shigellosis infections identified in Seattle (US) between 2017 and 2022 affected PEH [78]. Heavy precipitations are associated with a higher propagation in PEH to higher contamination of drinking water sources and overcrowding [105]. Bacteriemia may occur, although immunocompromise does not fully account for the severity of cases affecting PEH [102].

### International travellers to endemic regions

Travelling to zones where *Shigella* is endemic can pose health risks due to a lack of natural immunity and prophylaxis measures [108]. *Shigella* spp. are the cause of a significant number of diarrhoeal outbreaks during travel or military deployment in endemic countries [109–111]. In 2022, an outbreak caused by a multidrug-resistant (MDR) *S. sonnei* occurred among travellers from different European countries that were found to have stayed in the same facilities during a visit to Cape Verde [112]. Beyond the impact on the individual, contracting shigellosis while travelling can result in the introduction of antibiotic-resistant strains into new populations [113].

## Pathogenesis of *Shigella* spp

Blood and mucus in faeces are prominent clinical features of shigellosis that derive from extensive colonic epithelial damage and a highly inflammatory response. To comprehend these clinical manifestations, a thorough understanding of *Shigella* pathogenesis is crucial. Most data on *Shigella* pathogenesis have been elucidated from studies carried out in *S. flexneri*. However, notable discrepancies with *S. sonnei* have recently been highlighted. The absence of an animal model that thoroughly recapitulates human shigellosis restricts pathogenesis studies. Mice present limitations as they are poorly colonized by *Shigella* without prior antibiotic treatment [114]. Other animals such as rabbits and guinea pigs have been valuable to study *Shigella*-host interactions [115–119]. In recent years, the zebrafish model has emerged as a promising tool to study the pathogenesis of both *S. flexneri* and *S. sonnei* [120–122], and human intestinal organoids have shown great potential [123].

**Figure 1.**
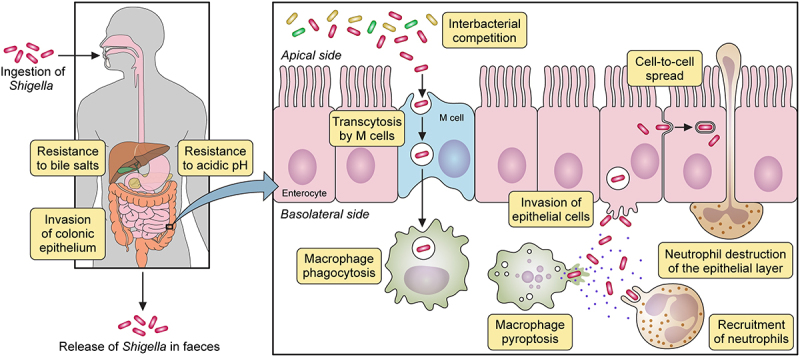
*Shigella* spp. pathogenesis. After ingestion and in its transit towards the intestine, *Shigella* must resist hostile conditions of the gastrointestinal tract e.g. stomach acidic pH and bactericidal action of bile salts. Once in the gut, *Shigella* competes with members of the commensal microbiota for space and resources. In the colonic epithelium, M cells transcytose *Shigella* from the apical to the basolateral side where it is internalized by macrophages. *Shigella* avoids vacuole-mediated macrophage degradation and induces their inflammatory pyroptotic cell death. Inflammation recruits neutrophils, which are efficient killers of *Shigella*. However, neutrophil migration damages the epithelial layer. Released from macrophages, *Shigella* invades epithelial cells through their basolateral side, multiplies intracellularly suppressing immune responses and cell death to extend the life of the infected cell. Bacteria then spread into neighbouring cells through actin-based motility. Finally, *Shigella* is released from the host through faecal matter.

The prevailing dogma for *Shigella* pathogenesis encompasses the following phases: bacterial cells reach the colonic epithelium and undergo transcytosis to the submucosa by M-cells, they are subsequently internalized by macrophages inducing their pyroptosis, they invade intestinal epithelial cells where they replicate in the cytosol, and they spread to neighbouring cells by actin polymerization. *Shigella* pathogenesis is summarized in Figure 1. These processes will be discussed in more detail below using research from *S. flexneri*. But where available, research on *S. sonnei* pathogenesis will be highlighted.

### *Shigella* type III secretion system (T3SS)

Type III secretion systems (T3SSs), present in a wide range of Gram-negative species, mediate bacteria-host cellular interactions [124]. The T3SS of *Shigella* is a fundamental element of its pathogenesis, as it enables the invasion of intestinal epithelial cells and the manipulation of host immune responses in various cell types. The system sequentially translocates effectors from the bacterial cytoplasm to the surrounding space or directly into target cells [125]. All genes encoding for T3SS structural components, and most regulators and effectors are found on the LVP pINV [15,56,126].

*Shigella* T3SS is a syringe-shaped complex that consists of a cytosolic sorting platform, a basal body spanning alongside the outer and inner membrane, and a needle that projects towards the extracellular space. The detailed structure of *Shigella* T3SS has been reviewed elsewhere [125,127]. The needle tip senses contact with the host and recruits IpaB and IpaC, also known as translocon proteins, which are secreted to form pores in the host membrane through which other effectors are delivered [128–130].

The expression of T3SS structural components and effectors is tightly regulated [131]. Under non-physiological temperatures (below 30°C), repressor histone-like nucleoid structuring protein (H-NS) represses expression of LVP-encoded transcriptional regulators VirF and VirB, and members of their regulons [125,132,133]. Apart from alleviating such repression, after colonziation of a human body, temperatures over 37°C cause a change in DNA topology that allow the production of VirF [134], which activates the transcription of *virB* [132] and adhesin *icsA* [135]. VirB counteracts H-NS-mediated silencing of virulence genes [136]. The presence of VirB leads to the upregulation of MxiE which activates the expression of late effectors and also relieves H-NS silencing [125,137,138].

### Phases of *Shigella* infectious cycle

#### Resistance to host barriers along the gastrointestinal tract

Upon entering in the human body, *Shigella* must tolerate the pH changes along their progression through the gastrointestinal tract towards the colon. In the stomach, it encounters highly acid conditions with a pH ranging from 1.5 to 2.8 [139]. However, as it continues heading to the small intestine, the pH becomes more alkaline (around 7.7). Ultimately, *Shigella* reaches its preferred site of infection, the colon, where the pH becomes slightly lower at around 6.4 [140]. Early studies demonstrated that *Shigella* can resist acidic environments for 2 hours at a pH as low as 2.5 [141]. Acid resistance is crucial for such a low bacterial dose to cause disease [142]. In addition, acidic conditions trigger virulence functions such as expression of the T3SS and biofilm formation [143].

*Shigella* mainly uses two pathways to counteract acidic conditions: the glutamate-dependent acid-resistance (GDAR) pathway and the oxidative pathway. The GDAR pathway is formed by decarboxylases GadA and GadB, and the glutamate/GABA antiporter GadC. The oxidative pathway works in the absence of glutamate and is inhibited by fermentative glucose-rich conditions [142,144]. Chaperones HdeAB also play a crucial role in the ability of *Shigella* to survive in these conditions [144,145]. The RNA polymerase RpoS, the transcriptional regulator SlyA and the RNA-binding chaperone Hfq have been found to be positive regulators of *hdeAB* and GDAR pathway genes [142,146,147]. Hfq regulates the expression of T3SS regulator VirB and a mutant exhibits reduced virulence and antibiotic resistance [148]. The transcriptomic study of this regulator revealed a genetic network that requires further characterization [149].

Bile, produced by the liver and secreted to the intestine, helps digestion and contains bile salts which facilitate bacterial clearance [150]. LPS and the AcrAB efflux pump play an important role in the resistance of *Shigella* to bile salts. Primary bile salt cholate and secondary bile salt deoxycholate promote biofilm formation (when glucose is present), which facilitates transit through the small intestine [151]. Bile salts also induce OspE1 and OspE2 expression and OspE1/2-mediated cell adherence and type 1 fimbriae expression in *S. flexneri* [151–153]. Deoxycholate promotes the recruitment of IpaB and IpaD to the tip of the T3SS complex [130,154] and induces IcsA-mediated adhesion and biofilm formation in *S. flexneri* [155,156]. However, deoxycholate downregulates IcsA expression and invasion in *S. sonnei* [157]. The different responses of *S. flexneri* and *S. sonnei* to bile salts and its relationship to cell invasion deserve further characterization.

Enteric pathogens must also penetrate the mucus layer that protects intestinal epithelial cells. The major component of mucus is the glycoprotein mucin [158]. *Shigella* both triggers host mucus production, a process accompanied by high inflammation [159–162], and degrades mucins [163,164]. *Shigella* protease Pic (from the family of Serine Protease Autotransporters of Enterobacteriaceae (SPATE)) presents mucolytic activity [165], promotes mucus secretion by specialized intestinal cells [160]. Mucus restricts oxygen diffusion and the relatively higher oxygen concentration at the epithelium surface compared to the lumen activates the T3SS promoting invasion [166].

#### Translocation to the basolateral side of the colonic epithelium

After overcoming the above-mentioned barriers, *Shigella* arrives at the apical side of the intestinal epithelium. While *Shigella* may adhere to the apical surface of intestinal epithelial cells [153,167] its ability to invade intestinal epithelial cells is greater through their basolateral side [162,168,169]. *Shigella* takes advantage of the function of microfold (M) cells to be transcytosed and access the basolateral side [115]. M cells are located in the follicle-associated epithelium (FAE). They work in the presentation of antigens from the intestinal lumen to the underlying subepithelial lymphoid tissue i.e. criptopatches, Peyer’s patches and other lymphoid follicles [170]. Transcytosis is not generally harmful to M cells as the bacteria remain within vacuoles [115], although some M cell cytosolic invasion and subsequent lateral spread to adjacent epithelial cells can occur [169].

#### Shigella-induced macrophage pyroptosis and interactions with neutrophils

Once at the basolateral side, *Shigella* encounters macrophages [168]. Other pathogens have evolved to replicate inside macrophages, but *Shigella* induces macrophage cell death to be able to reach its favourite niche i.e. colonic epithelial cells [171]. Following engulfment by macrophages, *Shigella* cells are sequestered within vacuoles. *Shigella* employs the T3SS to escape the vacuole and gain entry to the macrophage cytosol. Notably, vacuole escape also occurs during invasion of epithelial cells and the molecular mechanisms are thought to occur in a similar way in both cell types, although this requires further assessment. IpaB appears to be a major player in macrophage vacuole escape, as it creates pores in the phagosome membrane leading to its rupture [172,173].

**Figure 2.**
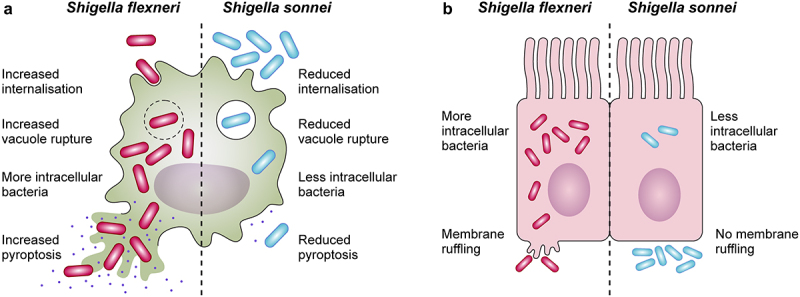
Differences in the interactions with macrophages and epithelial cells between *Shigella flexneri* and *Shigella sonnei*. (a) *Shigella flexneri* (depicted in magenta) shows increased internalization into macrophages mediated by its type 3 secretion system (T3SS), whereas the low internalization of *Shigella sonnei* (depicted in blue) depends predominantly on phagocytosis. *Shigella sonnei* also escapes from the shigella containing vacuole less efficiently in comparison to *Shigella flexneri*, which leads to a reduced cytosolic bacterial count. As a consequence, *shigella sonnei* induces less macrophage pyroptosis than *Shigella flexneri*. These differences are attributed to the double layer of O-antigen (found in the LPS and the group 4 capsule) in the cell envelope of *Shigella sonnei* (b) in contrast to *Shigella flexneri* infections, characteristic membrane ruffling is not observed upon invasion of epithelial cells by *Shigella sonnei*. The reduced invasion results in reduced intracellular growth of *shigella sonnei* compared to *Shigella flexneri*. Similar to macrophage interactions, these differences are linked to the presence of the double layer of O-antigen in *Shigella sonnei*.

Once in the cytosol, *Shigella* induces macrophage pyroptosis to evade antimicrobial action and allow invasion of epithelial cells [171,173–176]. Pyroptosis is an inflammatory form of cell death, driven by the assembly of inflammasomes and activation of caspase 1, that culminates with the release of proinflammatory cytokines interleukin-1β (IL-1β) and IL-18, the entry of excessive fluid into the cell, osmotic lysis, and leakage of contents [176–180]. Major inducers of pyroptosis in *Shigella* include T3SS constituents MxiH, MxiI [181,182] and cytosolic potassium decrease post-vacuole escape [183,184]. From a host perspective, pyroptosis is beneficial as it eliminates infected cells and exposes intracellular pathogens to an immune response [185]. Thus, the role of pyroptosis in *Shigella* infections is ambiguous, leaving questions about its impact for the host and the bacterium. Early observations described that *S. sonnei* caused less macrophage death than *S. flexneri* [186]. Recent findings highlight that *S. sonnei* induces reduced macrophage pyroptosis due to reduced internalization and vacuole escape, and induces less inflammation, likely due to shielding of the T3SS by its O-antigen. *S. sonnei* entry into macrophages appears to be by phagocytosis rather than bacterial-mediated invasion [187] (Figure 2a).

The highly inflammatory environment resulting from *Shigella*-induced macrophage pyroptosis exposes the bacterium to other immune components and cells e.g. neutrophils. Neutrophils resist induced pyroptosis better than macrophages and kill *Shigella* more efficiently by the action of reactive oxygen species (ROS), granule-contained antimicrobial molecules, and neutrophil extracellular traps (NETs) [188–191]. However, their migration to the infection site also contributes to damage since it disrupts the intestinal layer and favours further entry of *Shigella* to the basolateral side [192–194]. *S. flexneri* and *S. sonnei* have been shown to cause neutrophil death by necrosis [122,195]. Interestingly, the O-antigen of *S. sonnei* is crucial for protection from neutrophil clearance in zebrafish [122]. The study of interactions between *Shigella* and neutrophils is still in its infancy.

#### Adhesion and invasion of epithelial cells

Liberation from macrophages allows *Shigella* to contact the basolateral side of intestinal epithelial cells, where it binds different surface components. T3SS effectors IpaB, IpaC and IpaD bind fibronectin receptor α1β5 integrin [196]. IpaB can additionally bind hyaluronic acid receptor CD44 in cholesterol-rich domains [197,198]. Other components involved in adhesion include IcsA [155,157], MAM SSO1327 (exclusive to *S. sonnei*) [157], and the LPS [199].

*Shigella* invasion of epithelial cells is T3SS-dependent [126,200]. After contact with the host and IpaB-IpaC-mediated pore formation [201], effectors delivered into epithelial cells enable efficient attachment e.g. the IpaC-vimentin association [202], and manipulate host cytoskeletal actin architecture [203,204]. Interestingly, a microscopic analysis of *S. sonnei* interaction with epithelial cells did not show typical T3SS-mediated membrane protrusions and ruffling [187] (Figure 2b). On the basis of this result, the way *S. sonnei* invades epithelial cells requires further investigation.

#### *Shigella* intracellular survival in epithelial cells and cell-to-cell spread

Inside epithelial cells, like in macrophages, *Shigella* is first contained within vacuoles from where it must escape [172,205]. In order to survive intracellularly, *Shigella* must resist epithelial mechanisms of defence e.g. autophagy. Autophagy maintains cellular homoeostasis by recycling cellular components and eliminating intracellular pathogens in a lysosome-dependent manner [206]. NOD receptors detect cytosolic peptidoglycan and activate autophagy markers [207]. However, IcsA and IcsB can counteract autophagy by preventing bacterial recognition by such markers [208,209].

The cytosol of intestinal epithelial cells are *Shigella’s* preferred niche for replication. A proteomic study of intracellular *S. flexneri* revealed metabolic adaptations to this lifestyle. Intracellular iron levels are kept low due to its high toxicity, so *Shigella* up-regulates iron acquisition systems (Lut, Sit, FhuA and Feo) and the iron starvation-responsive protein Suf. As well, low intracellular oxygen levels make *Shigella* rely on mixed-acid fermentation (rather than respiration) using primarily pyruvate as a source of energy [210].

When the cytosolic niche of *Shigella* is eventually compromised, cell-to-cell spread is facilitated by actin polymerization by the autotransporter IcsA [211,212]. Double membrane vacuoles (DMVs) containing bacteria during intercellular spread can be degraded by IpgB1 [213]. As *S. sonnei* exhibits reduced invasion into epithelial cells [187,214] it will be interesting to evaluate whether it is also adapted to intracellular survival.

#### Prevention of epithelial cell death

Unlike the rapid pyroptosis induced in macrophages *Shigella* prevents cell death (apoptosis, necrosis and pyroptosis) in epithelial cells to safeguard its replicative niche [215–217]. Effectors VirA and IpgD promote degradation of pro-apoptotic factor p53 [218,219] and Spa15 prevents caspase 3 activation [220]. Necrosis is inhibited by NOD recognition of peptidoglycan, dampening BNIP3-CypD signalling [221]. *S. flexneri* induces less pyroptosis by changing its LPS from a hexaacylated to a tetraacylated form that is poorly recognized by the host [222]. Effectors OspC3, VirA, IpaJ, and IpaB reduce inflammatory responses [223–227]. Therefore, in epithelial cells *S. flexneri* induces a delayed necrotic cell death.

## Molecular mechanisms of *S. sonnei* virulence

**Figure 3.**
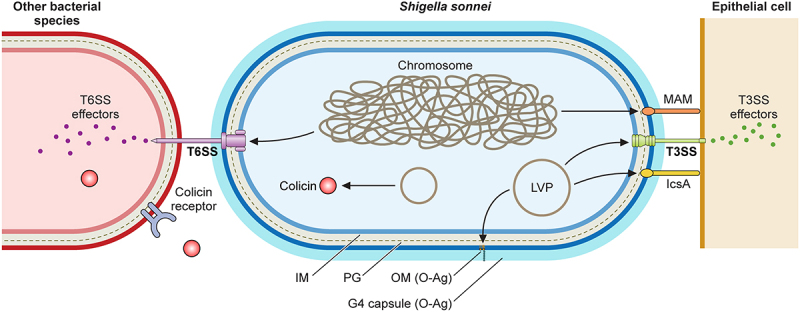
*Shigella sonnei* virulence factors. The large virulence plasmid (LVP) of *Shigella sonnei* plays a pivotal role in its virulence since it encodes for adhesin IcsA, the type 3 secretion system (T3SS) and the O antigen (O-Ag) synthesis cluster. The type 6 secretion system (T6SS), adhesin MAM and the capsule export locus are encoded on the chromosome, and colicins are usually encoded on other plasmids. During the invasion of epithelial cells, outer membrane proteins MAM and IcsA act as adhesins that facilitate T3SS-mediated invasion. The T3SS forms pores in epithelial cell membranes that enable the subsequent delivery of effectors that manipulate host cells. The interaction of *Shigella sonnei* with epithelial cells needs further characterization. *Shigella sonnei* has also evolved mechanisms to compete with other members of the intestinal microbiota, including a T6SS that requires close contact and colicins that target phylogenetically closely related bacteria and are released after bacterial lysis. *Shigella sonnei* produces a double O-Ag layer that is present in the LPS and the group 4 capsule and is involved in serum resistance.

The differences between *S. flexneri* and *S. sonnei* discussed throughout this review have served to re-evaluate the study of specific virulence factors in *S. sonnei*. Even though both species share a T3SS involved in cell invasion and host immune manipulation, invasion differs between the two. *S. sonnei* also has been found to possess unique virulence factors: the adhesin MAM SSO1327, a group 4 capsule, a unique O-antigen and mechanisms to compete with commensal bacteria. A schematic representation of *S. sonnei* virulence factors is shown in Figure 3. Uncovering *S. sonnei* virulence particularities is fundamental to tackle its escalating prevalence and to develop tailored prevention and control measures.

### Adhesins

Adhesion is key in the first stages of pathogen-host interactions. So far, two adhesins have been discovered in *S. sonnei*: multivalent adhesion molecule (MAM) SSO1327 encoded on the chromosome and IcsA encoded on the LVP.

MAMs (multivalent adhesion molecules) are outer membrane proteins broadly distributed among Gram negative bacteria that act in host recognition and attachment [228]. *S. sonnei* MAM SSO1327 is an adhesin that initiates, via fibronectin and phosphatidic acid, bacteria-host cell close contact needed for posterior cell invasion. Interestingly, in *S. flexneri* the MAM SSO1327 homologue is a pseudogene. A deletion mutant in *S. sonnei* SSO1327 presented less ability to adhere and invade epithelial cells and macrophages. In addition, it displayed low T3SS effector translocation and invasion of cells, despite secreting these effectors at wild type levels. Therefore, it has been suggested that this intimate contact, facilitated by SSO1327, activates the T3SS to translocate effectors into the target cell and allows subsequent invasion. Mutants lacking SSO1327 function cause less mortality in animal challenges [157].

The *S. sonnei* autotransporter IcsA acts synergistically with SSO1327 [157]. IcsA regulation differs in *S. sonnei* and *S. flexneri*. The bile salt deoxycholate upregulates IcsA-mediated cell adherence, invasion and biofilm formation in *S. flexneri* [151,155,229] but downregulates the expression of both SSO137 and IcsA, and consequently cell invasion, in *S. sonnei* [157]. These differences in the inducing stimuli may be key to understand early stages of S. *sonnei* infections.

### O-antigen

The LPS of Gram-negative bacteria is constituted by lipid A, core, and O-antigen. Unlike other *Shigella* spp. whose O-antigen synthesis genes are mostly encoded on the chromosome, those of *S. sonnei* are encoded on the LVP and were horizontally acquired from the environmental bacterium *P. shigelloides* [35,36,38,230]. The composition of this acquired O-antigen is unique as it contains two very infrequently encountered sugars: 2-acetamido-2-deoxy-L-altruronic acid and 2-acetamido-2-deoxy-L-fucose [230]. These sugars are also incorporated into the capsule of *S. sonnei* [214]. The genetic determinants for the synthesis of the O-antigen are encoded on the LVP but the export locus for capsule is encoded on the chromosome enabling genetic distinction [38,214].

*S. sonnei* O antigen plays a major role in virulence. In the zebrafish model, *S. sonnei* exhibits higher virulence than *S. flexneri* since a lower infective dose causes higher mortality. It was also found that a mutant deficient in O antigen synthesis exhibits a large attenuation of its infectivity. *S. sonnei* O-antigen confers resistance to neutrophil acidification [122]. Compared to *S. flexneri*, when in contact with macrophages *S. sonnei* induces less pyroptosis as it does not reach the cytosol efficiently. This is due to the double layer of O-antigen around *S. sonnei* cells that can act as a canopy to impair the action of the T3SS [187]. This is also seen in epithelial cells where mutants in which all O-antigen (LPS and capsule) are removed, and therefore the T3SS is more accessible, show greater invasion [214]. During interactions with macrophages a similar pattern occurs, so mutants where these layers are absent show greater internalization and cause increased pyroptosis [187].

### Group 4 capsule

Polysaccharides capsules are common bacterial virulence factors that act in protection against environmental stresses and evasion of the host immune system. They classify into four groups depending on genetic and biochemical factors [231]. Group 4 capsules utilize the O-antigen sugars to form a high molecular weight capsule, but how these sugars are anchored to the membrane remains to be identified [214]. *S. flexneri* O-antigen can only be found associated with the LPS and its capsule export operon is inactive [232].

The *S. sonnei* capsule plays an important role in resistance to serum complement [214]. Interestingly, *S. flexneri* regulates the length of its O-antigen, with short O-antigens resulting in higher levels of T3SS-mediated invasion and longer O-antigens mediating complement killing resistance [222,233,234]. *S. sonnei* has a shorter LPS in comparison but serum complement resistance can be mediated by the high-molecular weight capsule. Therefore the *S. sonnei* group 4 capsule may be an alternative to regulation of O-antigen length in *S. flexneri*. *S. sonnei* non-capsular mutants are less able to cause systemic infections, however they show enhanced capacity to invade intestinal epithelial cells, induce more inflammation, cytokine production and tissue damage. This has been attributed to the fact that IpaB effector of the T3SS is less accessible when the capsule is present [214]. The regulation of capsule production in *S. sonnei* and how it modulates the equilibrium between virulence and immune evasion deserve further study.

### Interference competition: T6SS and colicins

The human gut is one of the richest microbial ecosystems in terms of diversity, including more than 1000 different species, and of density, with an estimation of 10^11^ bacterial cells per gram of colonic content [235,236]. Gut flora plays a key role in nutrition but also constitutes a defence mechanism against microbial pathogens. To establish infection, gut pathogens must compete with resident commensal bacteria for nutrients and space [237]. Two mechanisms have been described in *S. sonnei* that would allow it to compete with resident gut bacteria: the type 6 secretion system (T6SS) and colicins.

More than 25% Gram-negative bacteria harbour T6SSs, including intestinal pathogens such as *Vibrio cholerae*, *Salmonella enterica* or *Yersinia enterocolitica* [238,239]. T6SS are syringe-like molecular machines that operate by injecting toxic proteins into other bacterial species, or in some cases into eukaryotes. A T6SS has been identified in *S. sonnei* but is absent in *S. flexneri*. The T6SS was shown to be active against both *S. flexneri* and *E. coli in vitro* and *in vivo* in a mouse model [240]. In terms of the interaction with eukaryotic cells, knockout T6SS mutants displayed no differences in virulence compared to wild type strains in a zebrafish model, suggesting that toxic effects are exerted only against other bacterial species [122]. The main function of this T6SS system would therefore be to provide *S. sonnei* with an advantage over other bacteria in colonizing the gut.

Colicins are a subset of bacteriocins that are produced by some bacterial strains/species to displace other closely related bacterial strains/species [241]. The killing of target cells does not require bacterial contact; colicins are secreted to the external medium by lysis and subsequently introduced into the target cells either by diffusion or by active transport. Bacteria carrying colicins can express and harbour these toxins because they also encode for immunity genes to prevent toxicity. The spB plasmid, which encodes for class E colicins, has been found in *S. sonnei* but not in *S. flexneri* [242]. Unlike the T6SS that is beneficial to the bacteria that encode it, colicins aid the general population by removing closely related species. A recent large-scale genomic analysis of *S. sonnei* strains identified genes encoding for colicins active against *E. coli* rather than the T6SS as being responsible for successful *S. sonnei* expansion [243]. These observations demonstrate that further studies are needed to characterize and elucidate the mechanisms that allow *S. sonnei* to displace the gut microbiota and establish infection.

## Treating *S. sonnei* infections: Current options and their limitations

A licenced and effective vaccine against *Shigella* is not yet available despite ongoing research into different approaches. Given that serotype-specific immunity occurs, one of the strategies where most progress has been made is the development of an O-antigen glycoconjugate vaccine. Another goal is to use conserved proteins that may give multivalent protection against different serotypes, simplifying production and cost [244]. Considering the current lack of a vaccine, antimicrobials are key to mitigate the impact of the disease. While rehydration therapy is typically sufficient for mild shigellosis, severe cases require antibiotics to minimize illness duration, severity, and transmission.

According to WHO guidelines, the fluoroquinolone ciprofloxacin is the primary antibiotic recommended for the treatment of shigellosis occurring with dysentery. Alternatives include azithromycin (macrolide), ceftriaxone, cefixime (both cephalosporins), co-trimoxazole (a combination of sulfamethoxazole and trimethoprim) and ampicillin [245,246]. In recent years, there has been a concerning rise in the isolation of *S. sonnei* strains exhibiting resistance to these antimicrobials ([247–249]. Some isolates are resistant to nearly all used antibiotics [250]. This trend has prompted the WHO and the Centers for Disease Control and Prevention (CDC) to categorize *S. sonnei* as a priority organism, demanding immediate attention for the development of innovative treatment lines [251,252].

Antibiotic resistance emergence in *S. sonnei* is associated with diverse mutations or the incorporation of drug-resistance plasmids acquired by horizontal transfer. Commensal intestinal bacteria act as a reservoir for mobile genetic elements present in *S. sonnei* [253]. *S. sonnei* lineage III, the most prevalent lineage, exhibits the highest degree of antibiotic resistance. The first resistances that appeared were against quinolones with mutations in the *gyrA* and *parC* genes common [254]. Resistance has also been reported against azithromycin through acquisition of the *mdhA* gene present in a plasmid [253]. Of particular concern is the emergence of resistance against alternative lines of therapy. The presence of B lactamases acting against cephalosporins such as ceftriaxone have also been described in *S. sonnei*, associated with the presence of the blaCTX-M gene [250]. Antibiotic availability to treat *Shigella sonnei* infections continues to decrease [255]. For example, a recent study carried out in China reported several outbreaks caused by *S. sonnei* isolates showing low susceptibility to fluoroquinolones, resistance to ceftriaxone, azithromycin and colistin (associated with a plasmid-encoded *mcr1* gene) [250]. Extensively drug-resistant (XDR) shigellosis in the US rose from 0% (2015) to 5% (2022) with *S. sonnei* being responsible for two-thirds of infections [256]. One of the main scientific challenges in *Shigella* research will be to find novel measures to treat these multi drug-resistant (MDR) and XDR infections without promoting resistance in other bacteria.

## Concluding remarks and future directions

*S. sonnei* is the major cause of bacillary dysentery in the developed world. No definitive answer has been found to link the association of economic prosperity and *S. sonnei* prevalence, and there remains much to explore about the biology of this emerging human-restricted pathogen.

Throughout this review, it has been discussed how *S. sonnei* has evolved distinctive features that set it apart from other species of the genus. It is distinguished by a very high person-to-person spread and high levels of antimicrobial resistance. There is an urgent need for effective treatment alternatives, as conventional first- and second-line antibiotics are increasingly rendering ineffective. The lack of an effective vaccine poses a significant challenge in mitigating this issue. Research into the species pathogenesis have brought to light discrepancies from the model bacterium *S. flexneri*, highlighting the fact that *S. sonnei* is less adapted to an intracellular lifestyle and interacts differently with host defences. In addition, *S. sonnei* encodes for specific virulence determinants (a unique O-antigen, the presence of capsule, a novel adhesin) and has mechanisms to displace other enteric bacteria. More studies are needed on the identification of novel virulence factors, their regulation and how they act altogether during the infection process. A well-established animal model that reproduces human shigellosis caused by *S. sonnei* would benefit this research.

Gaining deeper insights into its virulence determinants, antibiotic resistance mechanisms, and interactions with the host, will pave the way for new approaches to prevent and control *S. sonnei* infections.

## Data Availability

Data sharing is not applicable to this article as no new data were created or analysed in this study.
